# Adsorbent-Embedded Polymeric Membranes for Efficient Dye-Water Treatment

**DOI:** 10.3390/polym16111459

**Published:** 2024-05-22

**Authors:** Junaid Saleem, Zubair Khalid Baig Moghal, Snigdhendubala Pradhan, Ahsan Hafeez, Mohammad Shoaib, Johaina Alahmad, Gordon McKay

**Affiliations:** 1 Division of Sustainable Development, College of Science and Engineering, Hamad Bin Khalifa University, Qatar Foundation, Doha 5825, Qatar; 2 Center for Advanced Materials, Qatar University, Doha 2713, Qatar

**Keywords:** mineral-filled membranes, polymeric films, plastic waste, polyethylene and mixed-matrix membranes

## Abstract

Traditional bulk adsorbents, employed for the removal of dyes and metal ions, often face the drawback of requiring an additional filtration system to separate the filtrate from the adsorbent. In this study, we address this limitation by embedding the adsorbent into the polymer matrix through a process involving dissolution–dispersion, spin-casting, and heat-stretching. Selective dissolution and dispersion facilitate the integration of the adsorbent into the polymer matrix. Meanwhile, spin-casting ensures the formation of a uniform and thin film structure, whereas heat-induced stretching produces a porous matrix with a reduced water contact angle. The adsorbent selectively captures dye molecules, while the porous structure contributes to water permeability. We utilized inexpensive and readily available materials, such as waste polyethylene and calcium carbonate, to fabricate membranes for the removal of methylene blue dye. The effects of various parameters, such as polymer-adsorbent ratio, initial dye concentration, and annealing temperature, were investigated. Equilibrium data were fitted to Langmuir, Freundlich, Temkin, and Dubinin–Radushkevich isotherms. The equilibrium data were best represented by the Langmuir isotherm, with maximum adsorption capacity of 35 mg/g and 43 mg/g at 25 °C and 45 °C, respectively. The membranes can be regenerated and recycled with a 97% dye removal efficiency. The study aims to present a template for adsorbent-embedded polymeric membranes for dye removal, in which adsorbent can be tailored to enhance adsorption capacity and efficiency.

## 1. Introduction

Mineral-filled or heterogeneous polymeric films have become integral in the realm of separation applications [1,2,3]. These minerals are used as adsorbents in gas separation techniques [4,5,6,7] and as fillers in the hygiene market [8]. Specifically, calcium carbonate (CaCO_3_) emerges as the predominant filler in polymeric films within the hygiene industry [9]. The role of filler is limited to heat-stretching, in which it ensures uniform pores formation at the interface between the polymer and itself, imparting a microporous structure [10,11,12,13,14]. This unique design allows for the diffusion of water vapor while simultaneously acting as a barrier against liquid water [15]. The microporous configuration facilitates better air circulation and minimizes trapped moisture, playing a pivotal role in preventing skin rashes and irritations. However, the filler does not participate in the separation phenomena—adsorption or absorption.

In addition to its role as a filler, CaCO_3_ is widely used as an adsorbent for the removal of dyes and metal ions from wastewater [3]. Other commonly employed adsorbents are activated carbon, zeolites, and molecular sieves. The effectiveness of these adsorbents relies on two things: (a) high internal surface area and (b) the presence of active sites in the form of functional groups [16,17]. Although they are effective in the sorption of pollutants, their bulk form necessitates an additional filtration system to separate the filtrate from the adsorbent. This limitation could be overcome by employing a method that involves embedding adsorbent, such as CaCO_3_, into the polymer matrix.

This study focuses on the development of adsorbent-embedded polymeric membranes tailored for the efficient treatment of water contaminated with dyes. Our approach draws inspiration from the conventional utilization of CaCO_3_, which serves as a filler in polymeric films within the hygiene industry and an adsorbent for the removal of dyes and metal ions from wastewater. Unlike traditional filler applications, where CaCO_3_ is typically used in smaller quantities—ranging from 1–25% [4,15,18], often around 10%—our methodology distinguishes itself by employing a significantly larger amount, approximately 300% (three times the mass of the polymer). This increase aims to maximize the adsorption of dyes within the polymer matrix and sets our approach apart from conventional practices in filler applications.

The novelty in our approach lies in crafting a porous adsorbent–polymer composite membrane. This involves a sequential process of dissolution–dispersion, spin-casting, and heat-induced stretching. Most reported literature preferred extrusion–stretching routes in cases where semi-crystalline polymers—such as polyolefins—are involved [19,20,21,22]. Among polyolefins, polyethylene (PE) is the most chosen one due to its favorable attributes, which include low cost, processability, scalability, and versatility [12]. The integration of the adsorbent into the polymer matrix is facilitated by selective dissolution and dispersion. Simultaneously, spin-casting ensures the formation of a uniform and thin film structure [23,24], while heat-induced stretching results in a porous matrix. Notably, without the formation of a porous structure, this composite becomes unsuitable for dye-water separation, as the absence of pores renders the films impermeable to the passage of dye-water. The adsorbent selectively captures dye molecules, while the porous structure contributes to water permeability.

We opted for inexpensive and readily available materials, such as waste polyethylene and CaCO_3_, in the fabrication of membranes designed for the removal of methylene blue (MB) dye. MB is an organic dye with a cationic nature and is prevalent as a contaminant in industrial wastewater originating from various sectors, including plastics, textiles, cosmetics, and food manufacturing [1]. This holistic process demonstrates the efficiency and practicality of our membrane development methodology.

The purpose of this work was to evaluate the adsorption potential of adsorbent-embedded polymeric membranes using inexpensive and readily available materials—waste polyethylene and CaCO_3_—for MB dye. The equilibrium data of the adsorption isotherm were then studied to understand the mechanism of dye molecules onto the prepared composite membrane. This study presents a template for adsorbent-embedded polymeric membranes for dye removal, in which adsorbent can be tailored to enhance adsorption capacity and efficiency. These membranes hold significant promise for applications in industries such as textiles, dyeing, and wastewater treatment, where the removal of dyes from water is essential for both environmental sustainability and regulatory compliance.

## 2. Experimental Section

### 2.1. Materials

p-cymene, sourced from Njnq Bio-Tech Ltd. (Beijing, China), was used as a solvent for dissolving polyethylene without additional purification. Locally collected waste high-density polyethylene (HDPE) bottles were employed. Ultrahigh molecular weight polyethylene (UHMWPE) with a molecular weight ranging from 3–6 million g/mol was obtained from Sigma (St. Louis, MO, USA). CaCO_3_, procured from Sigma, served as an adsorbent without additional purification. Methylene Blue (MB) was supplied by Sigma. For membrane fabrication, a customized glass plate with a surface area of 25 cm^2^ was used as the solid substrate. Thin membrane films were annealed on a Heidolph hotplate (Schwabach, Germany).

### 2.2. Characterization Techniques

Tensile strength was measured using a friction/peel tester from Lloyd Instruments Ltd. (London, UK). Scanning electron microscope (SEM) images was captured with FEI Quanta650FEG (Hillsboro, OR, USA). Differential scanning calorimetry (DSC) determined the thermal behavior of hydrophobic thin films. X-ray diffraction (XRD) measurements were carried out using PANalytical Empyrean multipurpose XRD by Malvern Panalytical, Malvern, UK. Fourier-transform infrared spectroscopy (FTIR) was performed using the PerkinElmer Frontier instrument (Waltham, MA, USA). Thickness was measured with a micrometer and cross-referred with Deflesko FS3 PosiTector 6000 (Ogdensburg, NY, USA) using an iron metal base.

### 2.3. Membrane Preparation

A composite solution denoted as PE:CaCO_3_ (1:1), was prepared by dissolving 500 mg of UHMWPE and 500 mg of HDPE in 100 mL of p-cymene, followed by the addition of 1 g of dried CaCO_3_. The solution was heated to 130 °C for 20 min to obtain a completely dispersed composite solution. Simultaneously, a cleaned glass substrate was heated to 120 °C, and the hot composite solution was spin-coated onto the substrate. The spin coating process involved four steps: 300 rpm for 10 s, 700 rpm for 30 s, 1000 rpm for 60 s, and finally, 3000 rpm for 120 s. This spinning allows the formation of a thin membrane and also expels the solvent from the formed thin membrane. The glass substrate with the polymer composite was then placed on a hot plate at 130 °C for 2–5 min. The resulting polymer-composite membrane was peeled off and subjected to uniaxial stretching to obtain the desired thin film membranes. Similar procedures were followed to prepare PE:CaCO_3_ (1:2) and PE:CaCO_3_ (1:3) composite solutions, adjusting the amount of added CaCO_3_ accordingly.

### 2.4. Batch Equilibrium Studies

Methylene blue (MB) was used as an adsorbate. All solutions were prepared using distilled water. The choice of MB was based on its recognized strong affinity for adsorption onto solid surfaces [25]. The polymer-composite membranes, namely PE:CaCO_3_ (1:1), PE:CaCO_3_ (1:2), and PE:CaCO_3_ (1:3), were employed for the removal of MB from water at varying concentrations (1, 2, 5, 10, 20, and 100 mg/L). Each experiment was repeated under identical conditions. The concentrations of MB in the solutions before and after adsorption were determined using a double-beam UV–vis spectrophotometer. The amount of adsorption at equilibrium, *q_e_* (mg/g), was calculated by

(1) $$q_{e}=\frac{\left(C_{0}-C_{e}\right)V}{W}$$

where *C*_0_ and *C_e_* (mg/L) are the liquid-phase concentrations of dye at initial and equilibrium, respectively. *V* is the volume of the solution (L), and *W* is the mass (g) of dry adsorbent used. Equilibrium data were fitted to Langmuir, Freundlich, Temkin, and Dubinin–Radushkevich adsorption isotherms.

### 2.5. Membrane Regeneration

For regeneration, the membrane was immersed in methanol and stirred for 10–15 min or until the stains of dye were removed. This process dissolved MB in methanol, allowing the membrane to be regenerated and recycled.

## 3. Results and Discussions

The primary objective of incorporating the adsorbent into the polymer matrix is to augment adsorption capacity; thus, the stretching step exposes the adsorbent to the dye water. Moreover, in order to ensure optimal integration within the polymer matrix, as opposed to mere surface adherence, it is crucial to note that the selected adsorbents for this system should exhibit a density equal to or greater than that of the polymer–solvent matrix.

### 3.1. Morphology and Surface Properties

**Figure 1:**
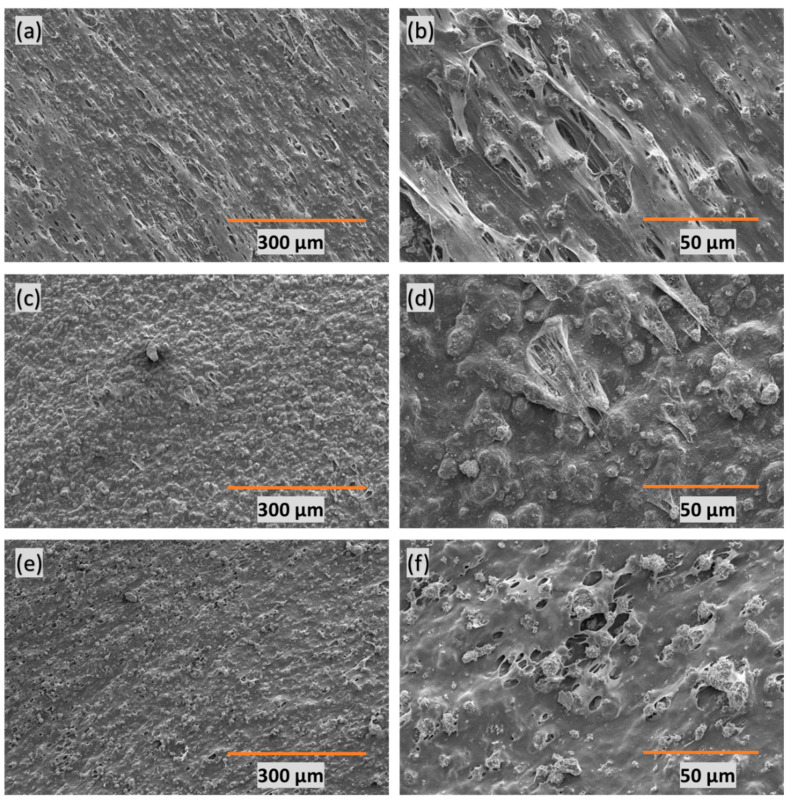
SEM images of membranes with different PE:CaCO_3_ ratios (**a**,**b**) 1:1, (**c**,**d**) 1:2, and (**e**,**f**) 1:3.

SEM was employed to analyze the porous structure of the adsorbent-embedded polymeric membrane. The presented images in Figure 1 depict varying PE:CaCO_3_ ratios—(a–b) 1:1, (c–d) 1:2, and (e–f) 1:3. In all three instances, micropores were formed through heat-stretching at the interface between the polymer and the adsorbent. The third case (1:3) reveals the highest amount of adsorbent distributed uniformly throughout the polymer matrix. The formation of pores facilitates the entry of dye water into the matrix, enabling efficient dye adsorption. Importantly, the heat-stretching procedure allows for in-depth adsorption of dye molecules, moving beyond mere surface adsorption. In essence, this unique design promotes adsorption in all three dimensions.

**Figure 2:**
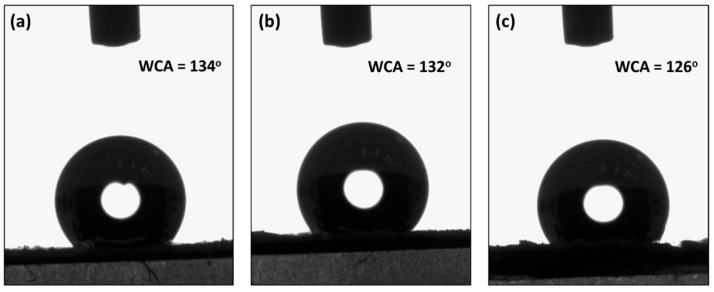
Contact angle of water on PE and CaCO_3_ (**a**) 1:1, (**b**) 1:2, and (**c**) 1:3 composite membranes.

Figure 2 illustrates the contact angle of the composite membrane. In order to improve dye-water penetration, the membrane should not be superhydrophobic (contact angle ~150°). Our spin-casting process yielded an extremely rough membrane surface with a water contact angle close to 148°, as reported previously [24]. However, the heat-stretching step facilitated in lowering of the water contact angle due to the heating of the polymer, which created oxygen moieties, as reported in our previous study [26].

### 3.2. Chemical Composition and Thermal Behaviour

**Figure 3:**
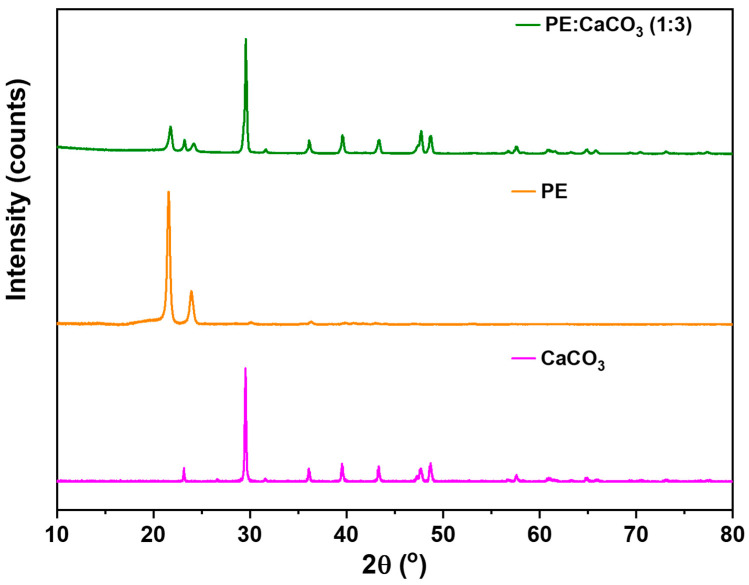
XRD patterns for PE and CaCO_3_ (1:3) composite membrane and pure PE.

To validate the integration of the adsorbent into the polymer matrix without altering the PE structure, we conducted a characterization of the membrane using XRD. Figure 3 presents the spectra of PE both with and without the adsorbent. The XRD analysis provides further confirmation of the adsorbent’s presence within the membrane structure.

The significance of this examination lies in addressing the potential release of the adsorbent during synthesis, particularly given its considerably higher density compared to the polymer. The polymer (PE) exhibits characteristic peaks at 22° and 24°, serving as reference points. All other identifiable peaks in the XRD spectrum are attributed to the adsorbent. Notably, the intensity of the PE characteristic peaks appeared smaller due to the relatively higher amount of the adsorbent.

**Figure 4:**
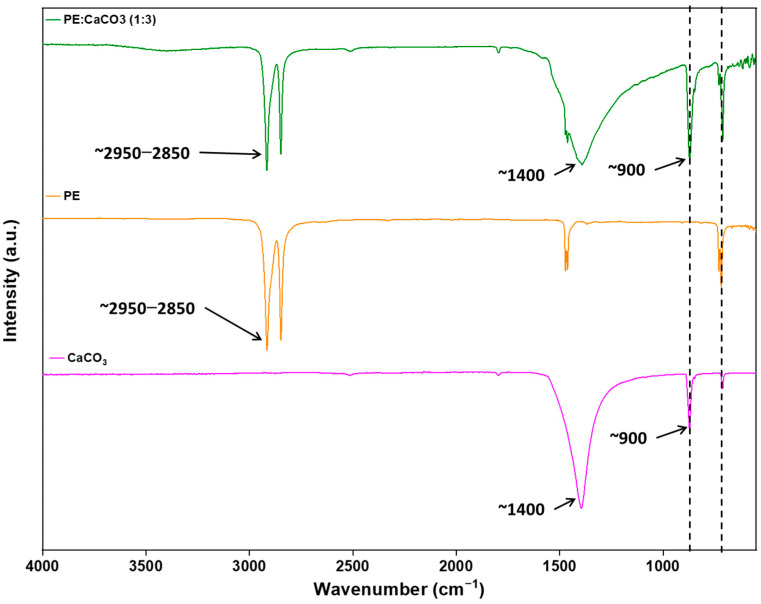
FTIR spectra of PE and CaCO_3_ (1:3) composite membrane, pure PE and pure CaCO_3_.

The incorporation of the adsorbent into the polymer matrix is further verified through FTIR. This confirmation is achieved by comparing the spectra of the composite membrane with those of pure PE, as illustrated in Figure 4. The adsorbent’s peaks are distinctly observed at around 1440 (asymmetric CO stretching), 873–898 cm^−1^ (out-of-plane deformation of carbonate), and 712 (OCO bending in-plane deformation vibrations of CaCO_3_), respectively, confirming the presence of CaCO_3_ and its successful integration with the polymer [18]. At 1500 cm^−1^, the PE peak merges with the peak of CaCO_3_.

Notably, the absence of new peaks in the spectrum suggests that there are no chemical changes occurring within the membrane. Instead, the interaction between the polymer and the adsorbent is predominantly intermolecular dispersion forces. This is supported by the observation that both PE and CaCO_3_ retain their characteristic peaks, signifying the preservation of their individual chemical identities within the composite structure.

**Figure 5:**
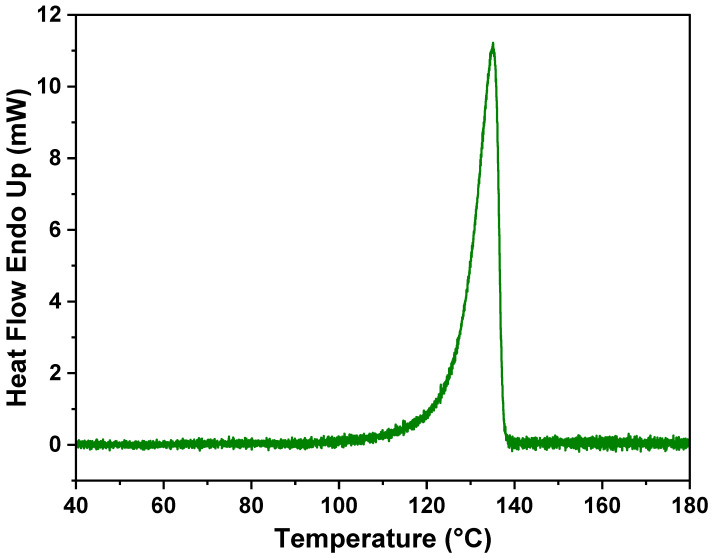
DSC spectra of PE and CaCO_3_ (1:3) composite membrane.

The thermal behavior of the membrane was examined by DSC and is presented in Figure 5. As observed, the melting point of the PE did not change due to the incorporation of the adsorbent, which further confirms that no new formulation or compound formed, and PE retains its characteristics.

### 3.3. Mechanical Properties

**Figure 6:**
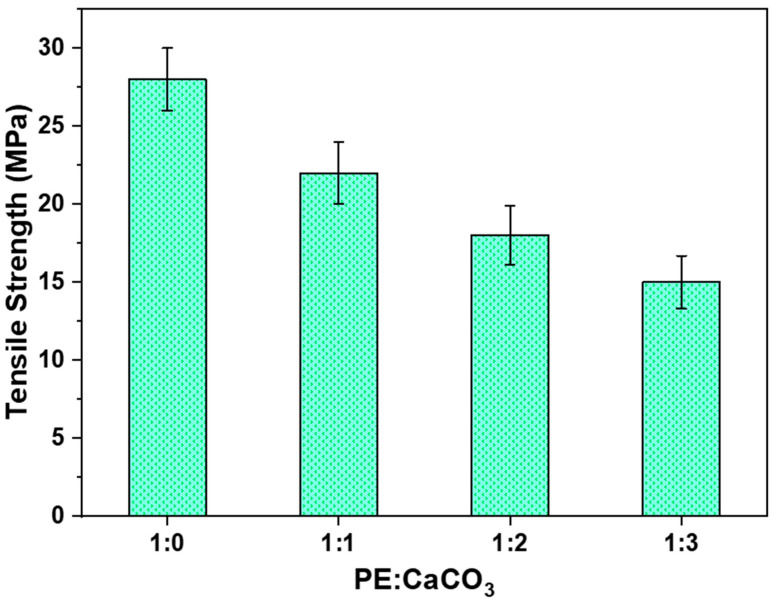
Tensile strength of the membranes with different PE:CaCO_3_ ratios.

Figure 6 presents the impact of the adsorbent on the tensile strength of the composite membrane. Four cases were studied with an increasing amount of adsorbent in the polymer matrix—1:0, 1:1, 1:2, and 1:3. As anticipated, the tensile strength decreases as the number of adsorbent increases in the polymer matrix, attributed to the increase in breakpoints within the structure.

The membrane exhibited the highest tensile strength (28 MPa) when the adsorbent was not added to its structure, whereas it showed the lowest value (14 MPa) when the polymer–adsorbent ratio was 1:3. The tensile strength of 14 MPa was sufficient for the application of the composite membrane in dye-water treatment. This is particularly crucial for the membrane’s regeneration and recyclability, as evident through recyclability studies that will be discussed further.

### 3.4. Adsorption Performance of Embedded Membrane

**Figure 7:**
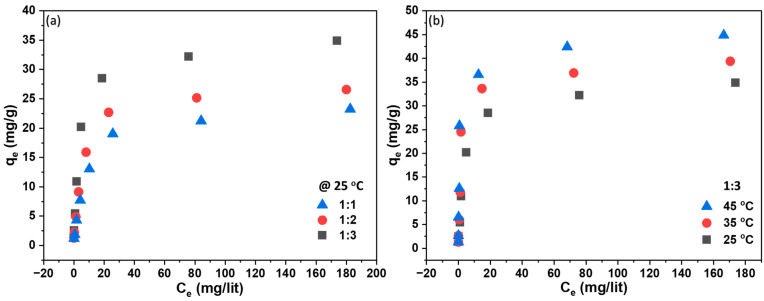
Plot of adsorption isotherm at equilibrium capacity: (**a**) with different membranes at 25 °C; (**b**) 1:3 membrane at different temperatures.

Figure 7a,b depicts the performance test of the adsorbent-embedded membrane, demonstrating a rise in adsorption capacity with an increasing amount of adsorbent and equilibrium concentration of the adsorbate. The maximum experimental adsorption values were 35 mg/g at 25 °C and 43 mg/g at 45 °C. In Figure 7b, the adsorption equilibrium is illustrated against various equilibrium MB concentrations against various temperatures. It was observed that for low MB equilibrium concentrations, the impact of temperature on adsorption equilibrium was not significant. However, at higher equilibrium concentrations (>60 mg/L), the adsorption equilibrium increased with a rise in temperature. This observed temperature-dependent adsorption trend aligns with the notion that elevated temperatures expedite the diffusion rates of dye molecules into the porous structure of the membrane. The heightened adsorption capacity may be attributed to chemical interactions between adsorbates and adsorbent, the creation of new adsorption sites, or an increased rate of intraparticle diffusion of MB molecules into the adsorbent pores at higher temperatures [25]. This improved diffusion allows for a more effective utilization of available adsorption sites, leading to increased adsorption. Furthermore, the temperature increase potentially enhances the chemical interactions between the surface functional groups of the membrane—facilitated through CaCO_3_—and the dye molecules. This, in turn, results in stronger adsorption bonds and increased adsorption efficiency.

### 3.5. Isotherm Study

**Figure 8:**
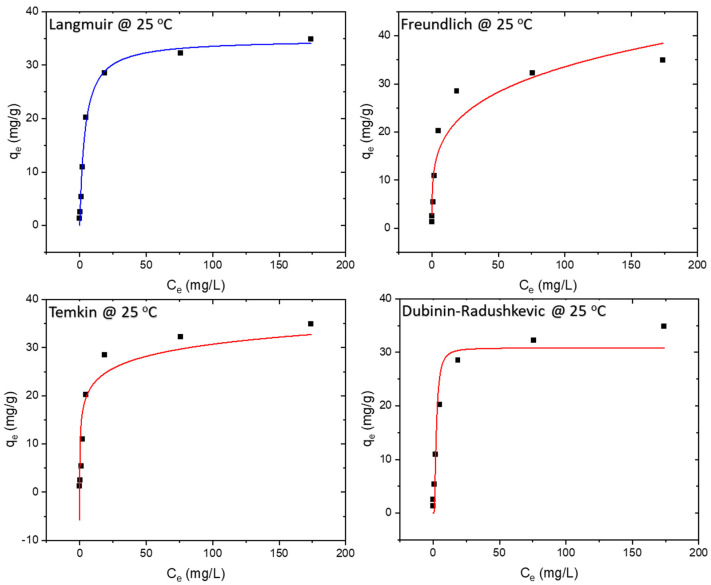
Isotherm models at 25 °C.

**Figure 9:**
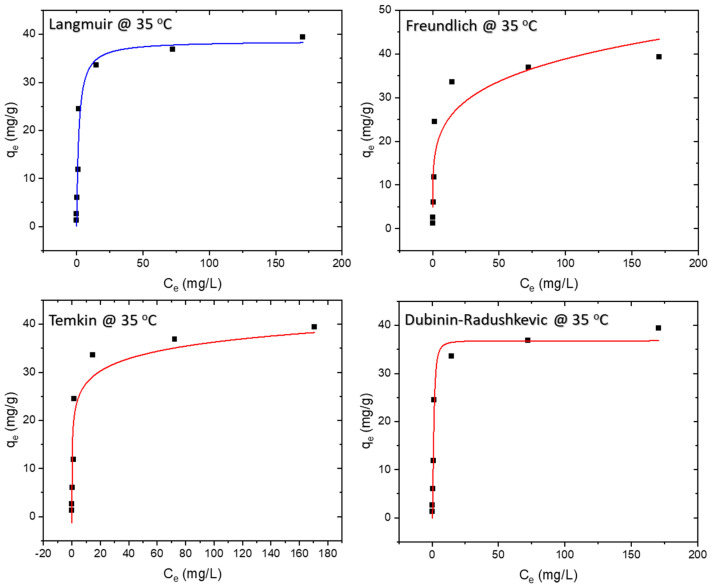
Isotherm models at 35 °C.

**Figure 10:**
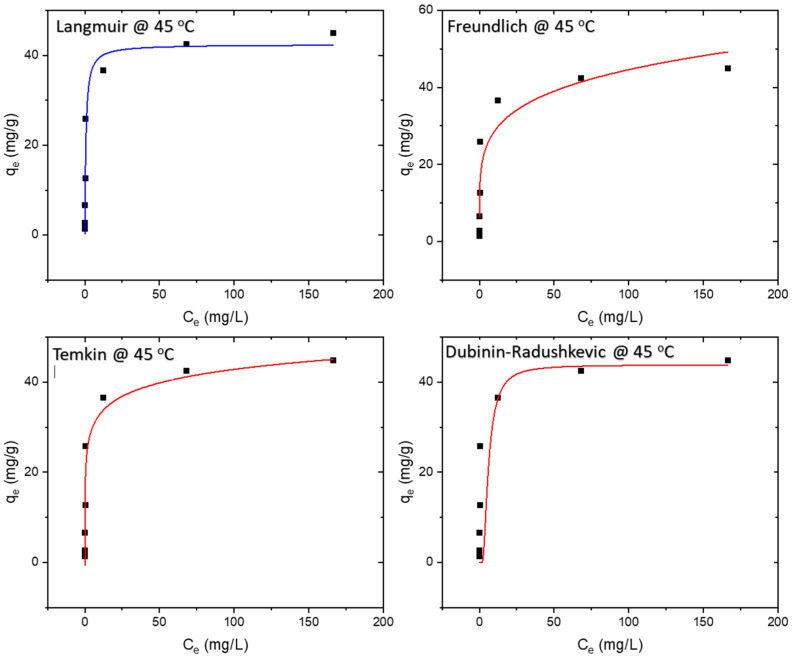
Isotherm models at 45 °C.

Adsorption isotherms are determined under equilibrium conditions. The experimental values were compared with four adsorption isotherms, namely Langmuir, Freundlich, Temkin, and Dubinin–Radushkevich. The equilibrium data were best represented by the Langmuir isotherm, with maximum theoretical monolayer adsorption capacity of 34.8 mg/g and 42.4 mg/g at 25 °C and 45 °C, respectively. The results of the isotherm study are presented in Figure 8, Figure 9 and Figure 10, whereas the model equations, parameters, and their values are tabulated in Table 1, Table 2 and Table 3.

The Langmuir isotherm assumes monolayer adsorption onto a surface with a finite number of uniform adsorption sites, involving no transmigration of adsorbate within the surface plane [27]. The Langmuir equation, as detailed in Table 1, features C_e_ as the equilibrium concentration of the adsorbate (mg/L) and q_e_ as the amount of adsorbate adsorbed per unit mass of adsorbent (mg/g). The R^2^ value of 0.993 indicates that the adsorption data for MB onto the membrane at all three temperatures—25 °C, 35 °C, and 45 °C adhered best to the Langmuir isotherm model.

The Freundlich isotherm, on the other hand, assumes heterogeneous surface energies, wherein the energy term in the Langmuir equation varies with surface coverage [27]. Represented by the equation in Table 1, the Freundlich isotherm involves C_e_ as the equilibrium concentration of the adsorbate (mg/L) and q_e_ as the amount of adsorbate adsorbed per unit mass of adsorbent (mg/g). The parameter ‘n’ provides an indication of the favorability of the adsorption process, with a value below one indicating a normal Langmuir isotherm and a value above one suggestive of cooperative adsorption.

The Temkin model (TM) assumes that adsorption heat as a function of all molecules’ temperature in the layer declines linearly rather than logarithmically due to the surface coverage increase [28,29]. The Temkin isotherm, detailed in Table 1, reflects these considerations.

For isotherms displaying a high degree of rectangularity, another widely used equation is the Dubinin–Radushkevich isotherm [27], also presented in Table 1.

**Figure 11:**
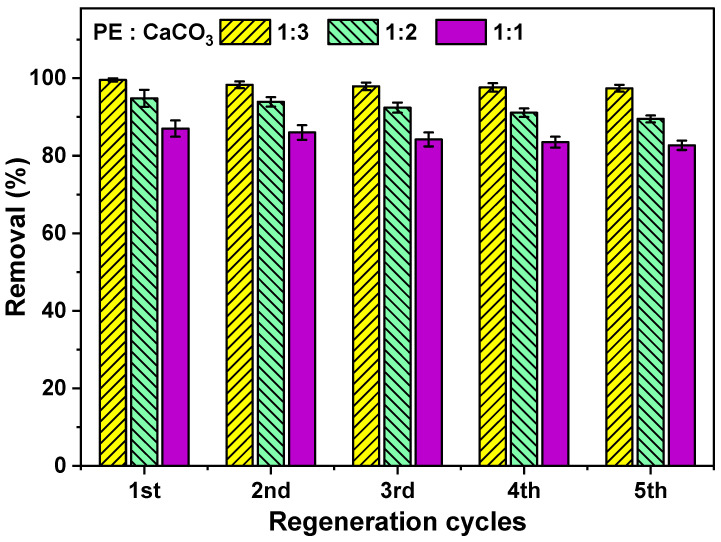
Regeneration cycles of PE:CaCO_3_ membranes for dye removal.

Figure 11 illustrates the regeneration cycles of the composite membrane. As demonstrated in the tensile strength section, the membrane exhibits sufficient strength to maintain its structure throughout the regeneration and recycling processes. In the regeneration experiment, it was observed that CaCO_3_ was susceptible to sonication, and the process could even break its particles, allowing them to dislodge from the polymer matrix. Therefore, it is recommended to employ normal stirring to effectively remove dye from the membrane. In recycling experiments, we observed a 97% dye removal efficiency even after the fifth cycle.

## 4. Conclusions

Our study successfully addresses the limitations associated with traditional bulk adsorbents by introducing an innovative approach in the form of adsorbent-embedded polymeric membranes. The incorporation of CaCO_3_ into a polyethylene matrix through a meticulous process involving dissolution–dispersion, spin-casting, and heat-induced stretching results in a porous composite membrane with significant advantages in dye-water treatment, especially in both selective dye molecule capture and water permeability. This porous configuration allows for three-dimensional adsorption, enhancing the overall efficiency of dye removal.

The adsorbent-embedded polymeric membranes exhibit promising efficiency in the removal of MB dye from water. The Langmuir isotherm fitting of equilibrium data reveals a maximum monolayer adsorption capacity of 34.8 mg/g at 25 °C and 42.4 mg/g at 45 °C. Notably, the membranes can be regenerated and recycled with a 97% dye removal efficiency, demonstrating their sustainability and practicality.

Moreover, our choice of inexpensive and readily available materials, such as waste polyethylene and CaCO_3_, adds to the cost-effectiveness and feasibility of the proposed membrane fabrication methodology. The membranes demonstrate significant promise for applications in industries such as textiles, dyeing, and wastewater treatment, addressing the critical need for efficient dye removal for environmental sustainability and regulatory compliance.

The adsorbent-embedded polymeric membranes presented in this study provide a template for an efficient approach to dye-water treatment, combining the advantages of adsorbent materials with the versatility of polymeric matrices. This research contributes to the ongoing efforts to develop sustainable and effective solutions for water treatment challenges in various industrial sectors.

## Figures and Tables

**Table 1:** Isotherm model equations with their respective parameters and values at 25 °C.

Model	Independent	Dependent	Equation	Parameters	Values
Langmuir Non-linear	C_e_	$q_{e}$	$\frac{\left(K_{L})(q_{m})(Ce\right)}{1+((K_{L})(Ce))}$	$q_{m}$	34.84136
				K_L_	0.2561
				R^2^	0.99329
Freundlich Non-linear	C_e_	$q_{e}$	${(K_{F})(Ce}^{1/n})$	n	4.06845
				K_F_	10.81772
				R^2^	0.91254
Temkin	C_e_	$q_{e}$	$\left(\frac{RT}{b_{T}}\right)ln(A_{T}Ce)$	b_T_	683.10816
				A_T_	47.25523
				R^2^	0.86343
Dubinin–Radushkevich	C_e_	$q_{e}$	$\left(q_{DR}\right)exp(-B_{DR}\cdot \varepsilon^{2})$	$q_{DR}$	30.82449
			$\varepsilon =RTln[1+\frac{1}{C_{e}}]$	$B_{DR}$	9.17907
				ε	738.05128
				R^2^	0.94599

**Table 2:** Isotherm models and parameters at 35 °C.

Model	Parameters	Values
Langmuir Non-linear	q_m_	38.63215
	K_L_	0.60493
	R^2^	0.96651
Freundlich Non-linear	n	4.86823
	K_F_	15.09673
	R^2^	0.88229
Temkin	b_T_	685.05074
	A_T_	163.04295
	R^2^	0.87783
Dubinin–Radushkevich	$q_{DR}$	36.80169
	B_DR_	3.28022
	ε	1234.62124
	R^2^	0.96695

**Table 3:** Isotherm models and parameters at 45 °C.

Model	Parameters	Values
Langmuir Non-linear	q_m_	42.41439
	K_L_	1.38943
	R^2^	0.95667
Freundlich Non-linear	n	5.17984
	K_F_	18.33436
	R^2^	0.90241
Temkin	b_T_	610.96986
	A_T_	198.08295
	R^2^	0.93852
Dubinin–Radushkevich	$q_{DR}$	43.82223
	B_DR_	4.46211
	ε	334.74559
	R^2^	0.61681

## Data Availability

The original contributions presented in the study are included in the article, further inquiries can be directed to the corresponding author.
